# Limited role of sessile acidophiles in pyrite oxidation below redox potential of 650 mV

**DOI:** 10.1038/s41598-017-04420-2

**Published:** 2017-07-10

**Authors:** Chang Liu, Yan Jia, Heyun Sun, Qiaoyi Tan, Xiaopeng Niu, Xuekun Leng, Renman Ruan

**Affiliations:** 1 National Engineering Laboratory for Hydrometallurgical Cleaner Production Technology, Institute of Process Engineering, Chinese Academy of Sciences, Beijing, 100190 China; 2 Key Laboratory of Green Process and Engineering, Institute of Process Engineering, Chinese Academy of Sciences, Beijing, 100190 China; 3 School of Chemistry and Chemical Engineering, University of Chinese Academy of Sciences, Beijing, 100049 China; 4 Wanbao Mining Limited, Beijing, 10053 China

## Abstract

Pyrite oxidation by mixed mesophilic acidophiles was conducted under conditions of controlled and non-controlled redox potential to investigate the role of sessile microbes in pyrite oxidation. Microbes attached on pyrite surfaces by extracellular polymeric substances (EPS), and their high coverage rate was characterized by scanning electron microscopy (SEM), confocal laser scanning microscopy (CLSM) and atomic force microscopy (AFM). The dissolution of pyrite was negligible if the redox potential was controlled below 650 mV (near the rest potential of pyrite), even though the bacteria were highly active and a high coverage rate was observed on pyrite surfaces. However, with un-controlled redox potential the rate of pyrite oxidation increased greatly with an increasing redox potential. This study demonstrates that sessile microbes play a limited role in pyrite oxidation at a redox potential below 650 mV, and highlight the importance of solution redox potential for pyrite oxidation. This has implications for acid mine drainage control and pyrite oxidation control in biometallurgy practice.

## Introduction

Pyrite (FeS_2_) is the most abundant sulfide mineral on earth and is found in most mining environments. It is frequently associated with coals and Cu, Co, Ni and Zn sulfide ores. Its oxidation is usually undesirable in commercial processes such as net acid generating bio-heaps, coal and petroleum refining, an environmental issue known as acid mine drainage (AMD). Consequently, understanding the dissolution mechanism and the role of bacteria in pyrite oxidation is necessary for commercial applications and environmental protection.

Great efforts have been made in recent decades to understand the mechanisms and kinetics of pyrite oxidation in both abiotic and biotic systems^1–9^. Currently, the thiosulfate pathway with a rate-determining reaction controlled by electrochemical processes is the most likely mechanism of pyrite oxidation in both abiotic and biotic systems^6, 10–15^. As expected for an electrochemically controlled process, the solution redox potential is an important parameter and is directly correlated with the oxidation rate^2, 16–20^. Sun *et al*.^20^ found that the pyrite dissolution rate decreased fivefold, if the redox potential was decreased by 100 mV and pyrite dissolution was less than 1.9% in 151 hours at 700 mV and 30 °C.

There has been a long-standing debate about the role played by microorganisms in pyrite bioleaching since the seminal study by Silverman and Ehrlich^21^. For the first time they explained the metal sulfide oxidation by two pathways: the direct and the indirect mechanism. However, the indirect mechanism has gradually been accepted^1, 10–13, 16, 22–28^. Sand *et al*.^23, 29^ further developed the idea of the “indirect mechanism” into “non-contact leaching” and “contact leaching”, by which the roles played by planktonic (free floating) and sessile (anchored) microorganisms were described, respectively. In “non-contact leaching”, pyrite is oxidized by ferric ions resulting in the generation of ferrous ions and various forms of sulfur compounds (Eq. 2). The ferric ions could be regenerated by planktonic iron-oxidizing microbes that act as electron sink re-oxidizing the ferrous ions (Eq. 1) causing a high solution potential:

1 $$4{{\rm{Fe}}}^{2+}+{{\rm{O}}}_{{\rm{2}}}+4{{\rm{H}}}^{+}\,\,\mathop{\longrightarrow }\limits^{{\rm{bacteria}}}\,\,4{{\rm{Fe}}}^{3+}+2{{\rm{H}}}_{{\rm{2}}}{\rm{O}}$$

2 $${{\rm{FeS}}}_{2}+8{{\rm{H}}}_{{\rm{2}}}{\rm{O}}+14{{\rm{Fe}}}^{3+}\to 15{{\rm{Fe}}}^{2+}+2{{\rm{SO}}}_{{\rm{4}}}^{2-}+16{{\rm{H}}}^{+}$$

Extracellular polymeric substances (EPS) are defined as “organic polymers of microbial origin which in biofilm systems are responsible for binding cells and other particulate material together (cohesion) and to the substratum (adhesion)”. “Contact leaching” occurs if sessile cells are adhering to the pyrite surface, with the extracellular polymeric substances (EPS) also serving as the reaction space for the oxidation of ferrous ions and dissolution of pyrite by ferric ions. However, this process is not completely understood^30^. The EPS are thought to contain ferric glucuronic acid complexes, and in addition cell-sized pits are observed on the pyrite surface, which are considered as evidence of “contact leaching”^31–33^. However, in these studies the solution redox potential is not reported, and the ferric concentration is high in the solution environment, which does not eliminate the effect of “non-contact leaching” on pyrite oxidation. In addition, the cell-sized corrosive pits could also form due to the chemical attack on the pyrite surface through the “non-contact leaching” process. Crundwell and Fowler compared the kinetics of pyrite oxidation with or without bacteria with the aid of an electrochemical apparatus controlling the redox potential at 800 or 850 mV^11, 13, 34^. They reported that the presence of bacteria on the pyrite surface altered the pH at the pyrite surface, hence an increase in the oxidation rate.

However, up to date, few studies have been conducted to investigate pyrite bioleaching at or below the rest potential of pyrite (650 mV). Under these conditions the effect of “non-contact leaching” on the pyrite oxidation is eliminated and only the “contact leaching” of pyrite by sessile bacteria is investigated. One of the difficulties inherent in this study was maintaining the solution redox potential below the rest potential of pyrite throughout the entire leaching process in the presence of highly active bacteria. Mixed iron-oxidizing mesophilic microorganisms were used in this study, since their enhanced adsorption characteristics were demonstrated in the previous studies^35, 36^. In order to keep the solution potential below 650 mV, the leaching solution was changed after 24 h leaching every day. The control experiments with different medium and non-controlled potential were performed to assist in demonstrating the role of sessile bacteria in pyrite bioleaching. The variation in pyrite dissolution and the solution-redox potential were recorded over time. Bacterial distribution on the pyrite surface and any surface changes resulting from bacterial leaching were investigated via scanning electron microscopy (SEM), energy dispersive spectroscopy (EDS), confocal laser scanning microscopy (CLSM) and atomic force microscopy (AFM). The conclusions provide useful theoretical support for heap bio-oxidation of pyrite and control of acid mine drainage.

## Results and Discussion

### Microbial community and activity

The use of a mixture of mesophiles, which had been collected from the acid mine drainage site of the Zijinshan Copper Mine in Fujian Province of China, may accelerate the dissolution of pyrite due to a strong attachment on the surface of pyrite^35^. The high-throughput sequencing method revealed the microbial community used in this experiment (Table 1). The most abundant bacteria were *Acidithiobacillus ferrooxidans*, and also small percentages of *Leptospirillum ferriphilum* and *Acidithiobacillus caldus*. The other detected genera included *Acidiplasma*, *Ferroplasma*, *Acidimicrobium*, *Sulfobacillus* and *Thiomonas* (data not shown). This mixture of acidophiles with high activity for iron and sulfur oxidation ensured the conduction of the leaching experiments.

**Table 1:** Composition of enrichment culture used in the leaching experiment analyzed by high-throughput sequencing (Species below 1% are not listed).

Species	Sequence number	Percentage (%)
*Acidithiobacillus ferrooxidans*	79148	80
*Leptospirillum ferriphilum*	8607	8
*Acidithiobacillus caldus*	6305	7
Others	2350	5

The average daily Fe^2+^ oxidation rate roughly reflects bacterial activity. During the 47 leaching days it amounted to 2.9 g/L·day and 2 g/L·day for Krouse medium (pH of 1.55) or MAC medium (pH of 1.55), respectively. These values are much higher than in some other reports^37^. Thus, the activity of our culture in the leaching experiments was high.

### Leaching experiments with and without microbes

The rest potential of sulfide minerals is an important electrochemical parameter to judge whether oxidation could occur or not. The rest potential of pyrite is about 650 mV and the value is slightly affected by solution conditions such as pH, presence of iron species, and presence of bacteria^38^. In order to understand the role of sessile bacteria (meaning anchored to the pyrite surface) in the leaching process, the solution redox potential was kept below the rest potential of pyrite to exclude the solution conditions that would render the electrochemical process spontaneous by chemical reaction. Before discussing the results in detail, it is necessary to explain, why pyrite coupons rather than pyrite powder with an increased surface area were used. Analysis by AFM, SEM and CLSM requires the use of polished flat surface coupons and the chosen particle size was close to industrial practices in Chile and Zijinshan^39^. In order to exclude the effect of the low surface area of pyrite coupons on a reduction of the pyrite-dissolution rate, experiments under non-controlled redox potential were performed with the same kind of pyrite coupons.

**Figure 1:**
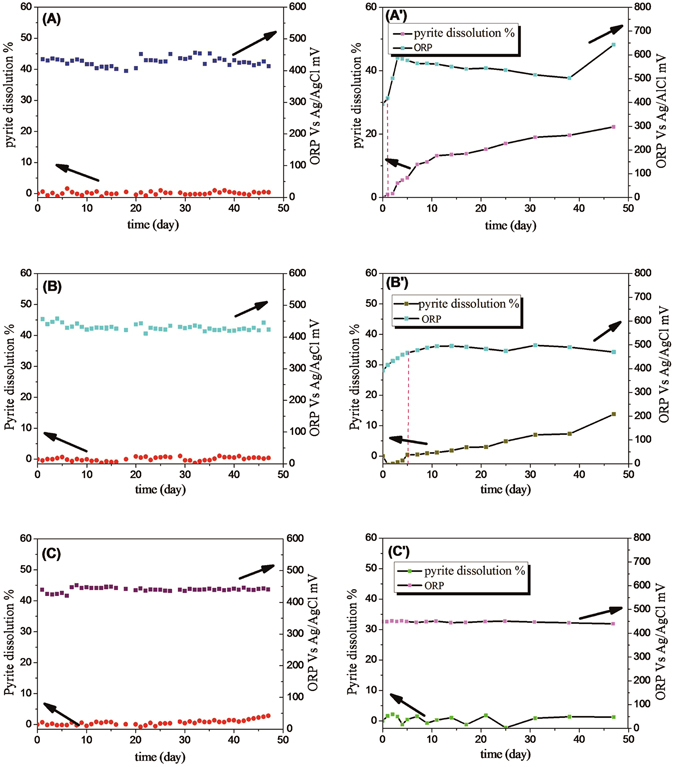
Pyrite dissolution and redox potential as a function of time. (**A**,**A’**) with/without control of the solution potential in a Krouse medium with an initial pH of 1.55. (**B**,**B’**) with/without control of the solution potential in a MAC medium with an initial pH of 1.55. (**C**,**C’**) control group without bacteria with/without daily change of the media.

In this work, the accumulative percentage for pyrite dissolution was calculated from the summation of the daily increase in the total species (TFe) concentration in solution. Cases with negative values can be attributed to a measurement error in the TFe concentration and to the almost negligible rate of leaching. The redox potential values shown in Fig. 1A–C were recorded after 24 hours of leaching, indicating that the solution redox potential was held below 650 mV for all leaching experiments with control of potential. As seen in Fig. 1A–C, after 47 days of leaching the accumulative percentage of pyrite dissolution remained below 3% regardless of the test conditions as long as the solution redox potential was held below 650 mV. Although the bacteria were identified to be highly active with different media of Krouse and MAC, the pyrite was hardly oxidized at the redox potential below 650 mV.

The pyrite dissolution and variation of solution potential for the bioleaching control tests are shown in Fig. 1A’–C’. The dissolution of pyrite coupons started when the solution redox potential approached and then exceeded the rest potential of pyrite at about 650 mV (marked by a dotted line). The test which was carried out at pH of 1.55 with the Krouse medium showed a striking decrease for the lag time with the redox potential quickly increasing to 780 mV at the second day. Afterwards, the redox potential slowly decreased to 710 mV and rose to 850 mV on the 47th day (Fig. 1A’). A similar trend was observed in the experiment carried out with the MAC medium at an initial pH of 1.55, but the solution potential was lower than that shown in (Fig. 1A’) and there was no peak in the solution potential during the later stages of leaching. The variation in the redox potentials of the two tests A’ and B’ may be attributed to the differences in the medium composition. One of the principal conclusions from these results is that oxidation of pyrite was negligible as long as the solution redox potential was kept below the rest potential of pyrite. When the solution redox potential was not controlled, an increased solution redox potential would result in a fast pyrite oxidation.

### Surface analysis of the interactions between microorganisms and pyrite

**Figure 2:**
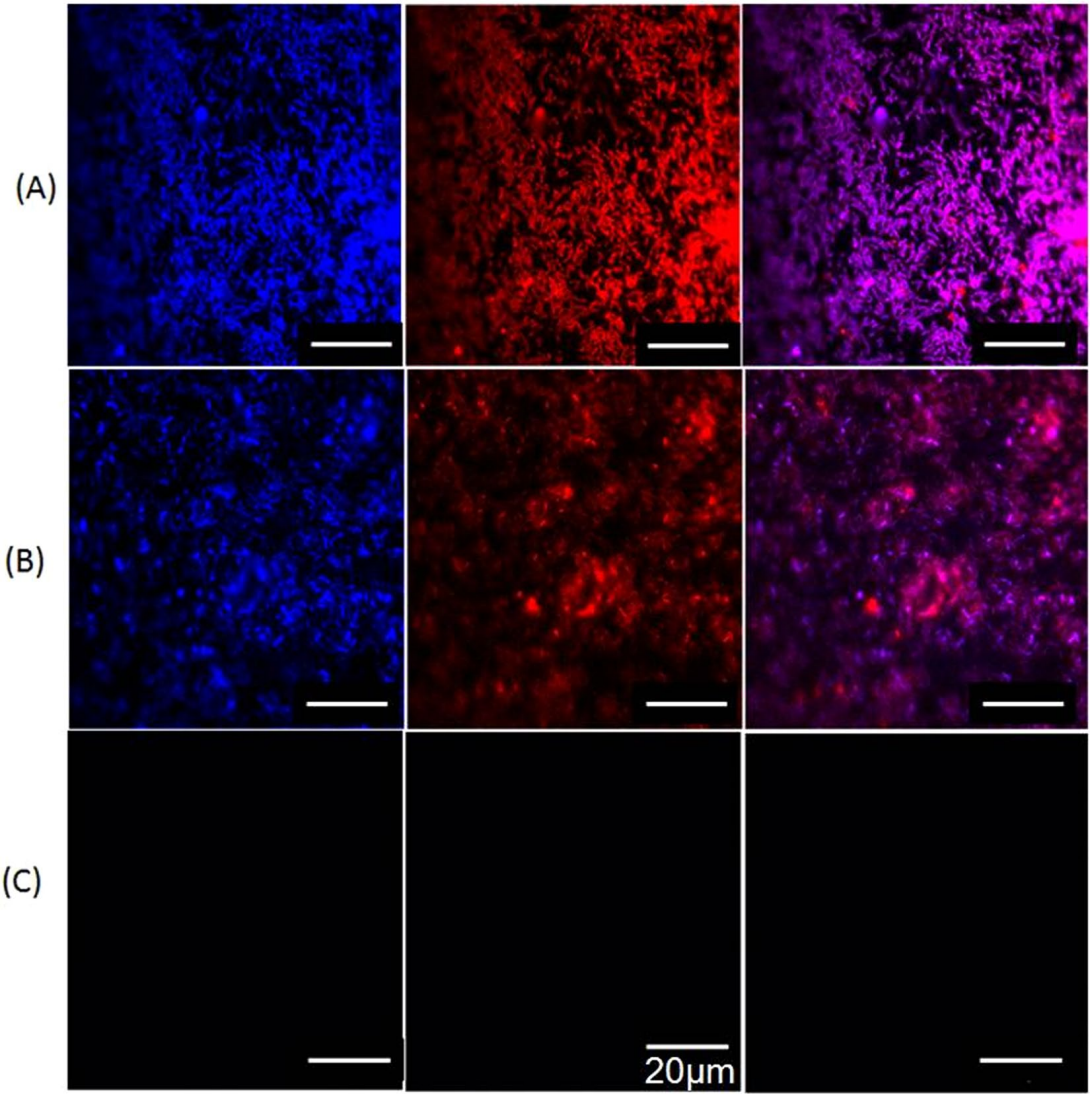
CLSM images of mixed mesophilic microorganisms colonizing pyrite coupons after 47 days bioleaching. Fluorescence of nucleic acid is visible in blue (first column) while lipids are visible in red (second column). An overlap of both epifluorescence images is presented in the third column. The bar represents a distance of 20 µm. Images in rows (**A**) show the coupon after leaching with Krouse medium at a pH of 1.55. Images in row (**B**) are for the coupon leached with MAC medium at a pH of 1.55. Images in (**C**) show the coupon of the control group.

The different coverage rates of bacteria on pyrite surfaces for different bioleaching conditions are shown in Fig. 2. The coverage rate of 75% with a Krouse medium (Fig. 2, row A) was larger than that with a MAC medium (Fig. 2, row B). Solution media (Krouse/MAC) with different nutrient composition have an effect on bacterial colonization. Even with a coverage rate of 75%, the dissolution of pyrite was negligible. This result suggests that at a redox potential below 650 mV, bacterial cell adhesion and formation of EPS did not result in pyrite dissolution. Furthermore, it suggests that the iron species concentration in the EPS space might be similar with the one in solution: the iron ions were in the ferrous form or even missing.

**Figure 3:**
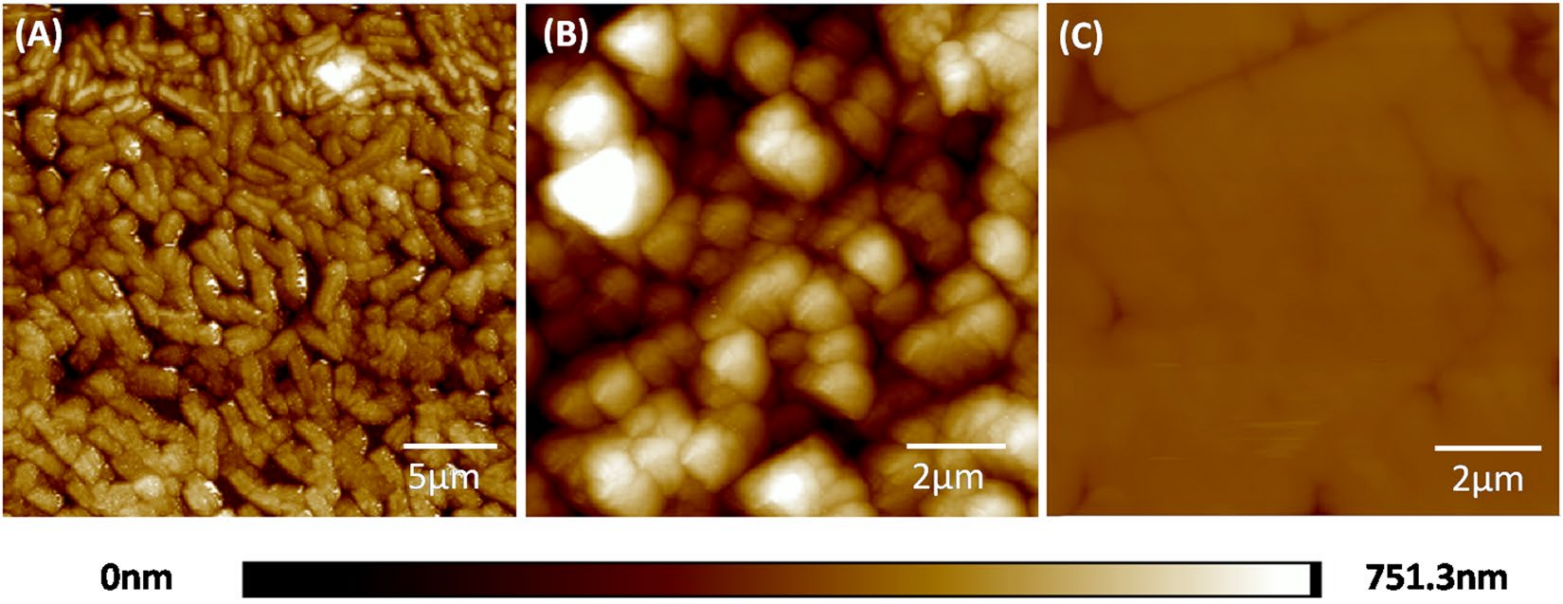
AFM images of mixed mesophilic microorganisms colonizing pyrite coupons after 47 days bioleaching. The pyrite coupon leached in Krouse medium at a pH of 1.55 (**A**). The pyrite coupon leached in MAC medium at a pH of 1.55 (**B**). The pyrite coupon of the control group (**C**).

Figure 3 shows the morphology of the pyrite surface and the distribution of cells on the coupons after the leaching process. In comparison to images obtained through AFM analyses, the three-dimensional information of a sample is provided. Interestingly, cells grown in the Krouse medium at pH 1.55 colonizing the pyrite surface were close-packed in one layer, while cells grown in the MAC medium arranged in multi-layers and overlapped with each other. Furthermore, the statistical thickness of the biofilm obtained with cells grown in the MAC medium was greater than that obtained with the Krouse medium. These observations suggest that the structure of the bacterial colonization is strongly influenced by the medium composition. The results and the discussion indicated that the formation of biofilm covered with bacteria regardless of single layer or multi-layer has a negligible effect on pyrite oxidation, if the solution redox potential was maintained below 650 mV.

**Figure 4:**
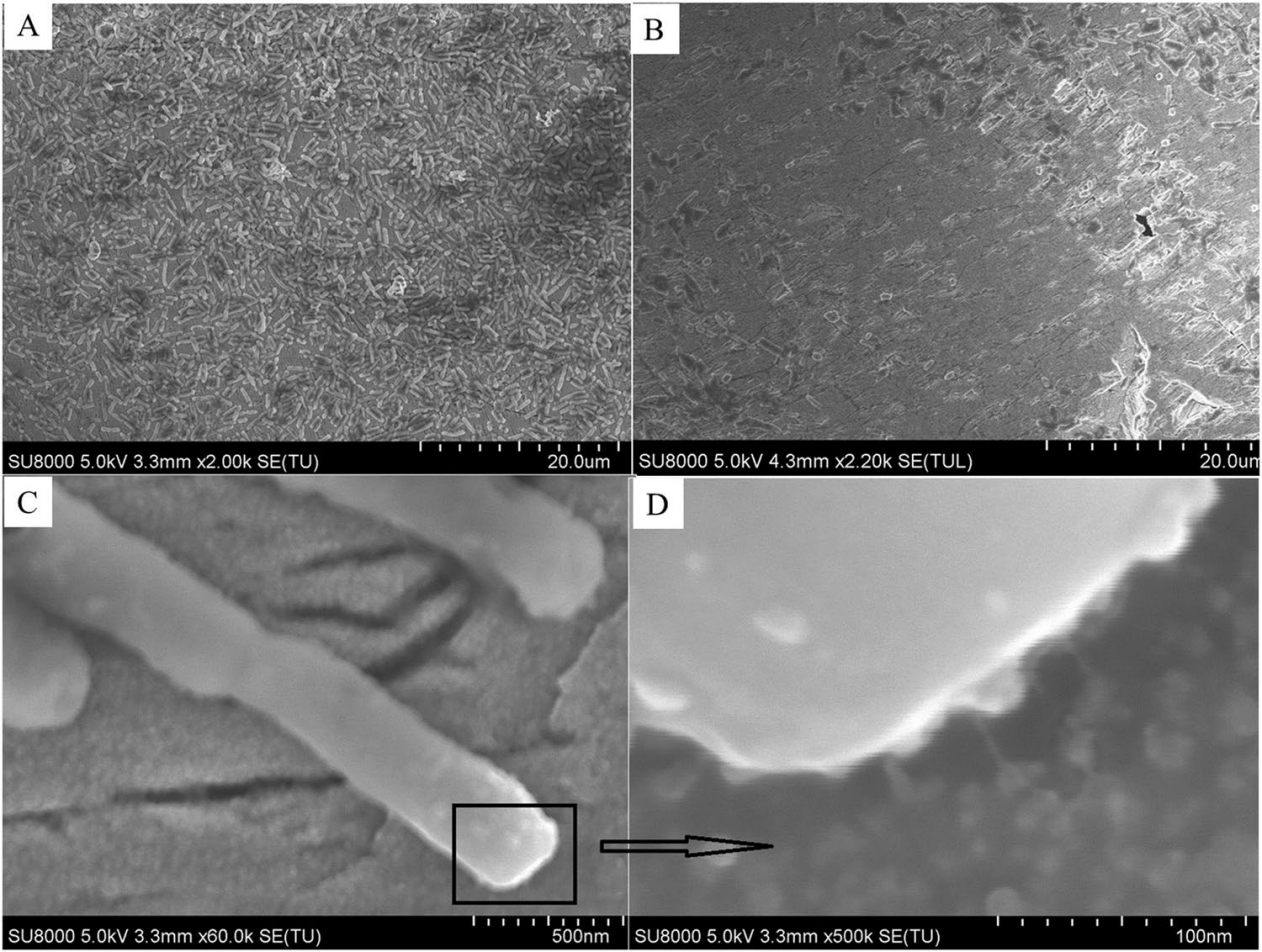
SEM images of pyrite coupons after 47 days leaching with Krouse medium at a pH of 1.55 (**A**) Macroscopic images of bacteria attached to a pyrite coupon. (**B**) Image of the pyrite surface after removal of the most strongly attached bacterial cells by ultrasonic treatment with 0.5% (w/v) Tween 80 solution. (**C**) Image of an individual cell attached to the pyrite surface. (**D**) Magnified image of the area shown in the square of (**C**). The smaller arrows in (**D**) indicate the locations of the pilis and EPS of a single cell. Scale of magnification shown at the bottom of each image.

The formation of the cell-sized corrosive pits could be the most important scientific basis to prove the existence of the “contact leaching” process^31–33^. However, Fig. 4A shows that the surface of the pyrite coupons was flat. This indicates that despite a large amount of bacterial cells being present on the pyrite surface, bacteria-mediated integral surface corrosion of pyrite is absent. To investigate the points of contact between bacteria and pyrite in detail, a typical single cell attached to the pyrite surface was chosen (Fig. 4C). Figure 4D is a magnified image of the square area shown in Fig. 4C, and from Fig. 4D the cell was observed to adhere to the pyrite through pili and EPS. In addition, there were no obvious corrosion pits at the contact edge between bacteria and pyrite surface. To further explore the pyrite surface under the biofilm, the biofilm and attached cells were removed using ultrasonication. The result is shown in Fig. 4B. After the biofilm was removed, most of the mineral surface was found to be flat and so called cell-shaped “pits” were not present. All results confirm that the sessile bacteria could not oxidize pyrite, if the solution redox potential was controlled below 650 mV.

This work offers useful information for controlling acidity generated during heap bioleaching. The solution redox potential is a critical factor that could act as a switch to control pyrite oxidation. In a real-life mining situation, the redox potential in a pregnant leach solution (PLS) and at ore surface are strongly affected by microbial activity and quantity. Another result of this work (data not shown in this paper) certified that microbial colonization of pyrite was found to be greatly inhibited beyond a certain acidic pH (1.55). This is in agreement with the industrial practice of Zijinshan mine, where the pH of the PLS is found to be 0.92, and in these highly acidic systems bacterial concentrations are reduced to 10^4^ cells/mL^40^. According to the results and the discussion above, it is suggested that if the redox potential of the leaching solution is controlled below the rest potential of pyrite, then acid generation from the oxidation of pyrite will be greatly inhibited, and the environmental problem of acid rock drainage will be reduced.

## Conclusions

Bio-oxidation of pyrite in a solution with a redox potential below 650 mV was investigated with a novel method. Under these conditions, and after eliminating the “non-contact chemical attack” present in solution, oxidation (and hence dissolution) of pyrite was negligible although more than 70% of the pyrite surface was covered by bacteria. These results suggest that sessile bacteria are of limited impact on pyrite dissolution, if the redox potential is kept below 650 mV. The flat surface of pyrite observed after removing the biofilm further confirmed that the sessile bacteria and EPS played a limited role in pyrite oxidation, if the solution potential was controlled below 650 mV. For practical applications to reduce discharge of acid mine drainage (AMD), this study suggests that acid generation can be controlled only if the leaching solution redox potential is kept below 650 mV. However, more detailed studies for evaluating the processes that occur at the interface between the bacterial EPS and pyrite should be conducted.

## Methods

### Pyrite preparation

Pyrite coupons (sized approximately 1 × 1 × 0.3 cm) were cut from natural pyrite crystals purchased from Tongling, Anhui Province of China. The coupons were manually polished using diamond suspensions with grain sizes of 3 µm and 1 µm. The sample coupons were then cleaned with ethanol in an ultrasonic bath and subsequently boiled in 6 N HCl to sterilize and remove bacteria and iron oxides from the mineral surface^41^. After treatment with HCl, the coupons were washed with deionized water and dried under N_2_ for 24 h.

### Leaching experiments

Leaching experiments were performed in 100 mL Erlenmeyer flasks, each containing 50 mL Krouse or Mackintosh (MAC) medium, a pyrite coupon (≈1.8 g) and an initial cell number of 5 × 10^7^ cells/mL (control experiment without addition of bacteria). The flasks were shaken with 120 rotations per minute (rpm) at 32 °C. The modified Krouse medium contained K_2_HPO_4_ (17 mg/L), MgCl·6H_2_O (102 mg/L), NH_4_Cl (53 mg/L), and the modified MAC medium contained KCl (52.2 mg/L), KH_2_PO_4_ (41 mg/L), MgSO_4_ (49.3 mg/L), CaCl_2_ (6.7 mg/L) and Na_2_MoO_4_·2H_2_O (9.7 mg/L). The pH was adjusted to 1.55 by 98% H_2_SO_4_. These two media were used to ensure a good growth of microbes and minimize the formation of jarosite.

For experiments with a control of the solution potential, in order to keep the solution potential below 650 mV (throughout this paper potentials are quoted with reference to the standard hydrogen electrode, SHE), the initial redox potential of the leaching medium was adjusted to about 600 mV ( ± 5 mV) daily by adding a total iron concentration of 10 g/L consisting of 2.9 g/L of ferric ion and 7.1 g/L ferrous ion. The leaching solution was substituted with fresh medium after 24 h leaching. Thus, the pyrite coupon was continuously immersed in the “low potential” solution. Measurement of solution potential and sampling were completed before solution changing. The experiments without control of solution potential were performed to exclude the effect of low surface area of pyrite coupons on reduction in the pyrite dissolution rate, and further test the effect of solution redox potential on pyrite dissolution. For experiments under non-controlled potential, the experimental conditions were not changed except for the leaching solution volume being 250 mL. Every flask was sampled at days 0, 5, 7, 9, 11, 14, 17, 21, 25, 31, 38 and 47 by removing 1 mL aliquot from the leaching solution rather than changing the solution daily. The solution potentials were recorded at the same time. The control experiments were not inoculated and were conducted under the same conditions except for the initial potential of 650 mV.

After sampling, the TFe and Fe^2+^ concentrations and the total cell numbers were measured. The TFe concentration was measured using inductively coupled plasma-optical emission spectrometry (ICP-OES), and the Fe^2+^ concentration was determined by titration with potassium dichromate^42^. The difference between TFe and Fe^2+^ concentrations provides the concentration of Fe^3+^ions. The pH was monitored using a Mettler Toledo SG8 pH electrode. The redox potentials were measured using a FermProbe Pt electrode with an Ag/AgCl reference electrode (3.8 M KCl).

### AFM analyses

Atomic force microscopy (AFM) was used to study the cell morphology and distribution of the cells present on the surface of pyrite coupons. After the leaching process was completed, the pyrite coupons of both biotic and abiotic assays were collected, and air-dried at ambient temperature in a desiccator for 1 h prior to imaging. The AFM analysis of a pyrite coupon surface was performed using a Fastscan Bio Bruker instrument. The silicon cantilever with a free resonance frequency ranged from 90 to 115 kHz and a force constant between 1.08 and 2.03 N/m was chosen for scanning. For AFM imaging, tapping mode was applied. The scan rate ranged from 0.5 to 1 Hz.

### Cell staining and CLSM analyses

After AFM analysis, the pyrite coupons were rinsed with redistilled water and PBS (pH 7.2 phosphate buffer) solution to further remove planktonic cells and the acidic medium. The biotic and abiotic residual coupons were sequentially stained in the dark for 30 minutes with DAPI solution (0.01%4′, 6-diamidine-2-phenylindole) and Nile Red (NR; Sigma) fluorophores, for the fluorescence detection of EPS (lipids) and genetic material (DNA), respectively. Subsequently, PBS and acetic acid (5% in ethanol) were used for washing and dissolving the DAPI and Nile Red stains, respectively. Thereafter, the stained coupons were mounted on glass slides and observed with a laser scanning module (LSM 710 Carl Zeiss® Jena) using a diode laser at 405 nm and a band-pass filter (410–520 nm) and a He-Nelaser at 543 nm with a 560-nmlong pass filter for acquisition of the fluorophore signal and subsequent surface mapping by light reflection at constant laser energies and detector settings. The microscope was operated with Zen 2010 (Zeiss®) software. Image quantification was carried out with Image J. For quantification of the cell-covered pyrite coupon surface, manually thresholder 8-bitimages images were used^37^.

### SEM-EDS analyses

After 47 days of leaching, the distribution of cells on pyrite surfaces was examined by SEM-EDS (Scanning Electron Microscope-Energy Dispersive Spectrometer). The coupons were fixed with 3% glutaraldehyde in the same PBS solution (pH 7.2 phosphate buffer) at 4 °C overnight. The coupons were then rinsed with PBS and dehydrated by immersion in 50%, 75% and 99% ethanol solutions for 10 minutes each. After dehydration, the coupons were air dried in a fume hood for 2 hours at room temperature. Afterwards, the coupons were coated with platinum by electro-deposition and examined by SEM (JSM-7001, Japan) coupled with EDS (INCAX-MAX) at 15 kV accelerating voltage.

### Bacterial cultivation and taxonomic classification

The mixed mesophiles used in the bioleaching experiments were obtained from the acid mine drainage site of the Zijinshan Copper Mine in Fujian province of China. For screening the iron-oxidizing bacteria, the enrichment culture was cultivated using 9 K medium, containing 3.0 g/L (NH_4_)_2_SO_4_, 0.1 g/L KCl, 0.5 g/L MgSO_4_·7H_2_O, 0.5 g/L K_2_HPO_4_, 0.01 g/L Ca(NO_3_)_2_ and 44.7 g/L iron (II) sulfate at an initial pH of 1.55 in an orbital incubator-shaker at 31 °C. After several times of subculture and activation, the inocula were harvested in the stationary phase and treated as follows.

The culture solution was filtered through Whatman filter paper (No. 1) to remove precipitates like jarosite. The filtrate containing cells then underwent centrifugation at 9000 rpm for 15 minutes to pellet the cells. After washing the residue twice with sterilized 9 K solution (adjusted to a pH of 1.55 without FeSO_4_·7H_2_O), the cell pellet was re-dispersed with an appropriate amount of 9 K solution to form the inocula. The concentration of cells in the inocula was determined by direct cell counting prior to addition to the flask for the bioleaching experiment.

The solution sample was filtered through a Millipore filter (0.22 μm; 10 mm diameter). The bacteria on the filter papers were then transferred into centrifuge tubes, and centrifuged at 14,000 × g for 10 min to collect the cells. The community DNA was extracted using the FastDNA Spin kit (Bio 101, USA) according to the manufacturers protocol. Primers F515 (5′-GTGCCAGCMGCCGCGGTAA-3′) and R806 (5′-GGACTACVSGGGTATCTAAT-3′) were used to amplify the bacterial and archaeal 16 S rRNA genes V4 hyper variable region^43^. The purified PCR products were mixed in equal concentrations and sequenced using Miseq (Illumina, USA) following the manufacturers instructions. Sequences were clustered into operational taxonomic units at a similarity rate of 97% using the furthest neighbor algorithm by ClustalW. Taxonomic classification of each phylotype was determined using the Ribosomal Database Project with a minimum confidence interval of 80%.

## References

[1] Boon M, Heijnen JJ (1998). Chemical oxidation kinetics of pyrite in bioleaching processes. Hydrometallurgy.

[2] Bouffard SC, Rivera-Vasquez BF, Dixon DG (2006). Leaching kinetics and stoichiometry of pyrite oxidation from a pyrite–marcasite concentrate in acid ferric sulfate media. Hydrometallurgy.

[3] Brunner, B. *et al*. Different isotope and chemical patterns of pyrite oxidation related to lag and exponential growth phases of *Acidithiobacillus ferrooxidans* reveal a microbial growth strategy. *Earth and Planetary Science Letters* **270** (2008).

[4] Descostes M, Vitorge P, Beaucaire C (2004). Pyrite dissolution in acidic media. Geochim. Cosmochim. Acta.

[5] Gleisner M, Herbert RB, Kockum PCF (2006). Pyrite oxidation by *Acidithiobacillus ferrooxidans* at various concentrations of dissolved oxygen. Chemical Geology.

[6] Holmes PR, Crundwell FK (2000). The kinetics of the oxidation of pyrite by ferric ions and dissolved oxygen: an electrochemical study. Geochim. Cosmochim. Acta.

[7] Ma Y, Lin C (2013). Microbial oxidation of Fe and pyrite exposed to flux of micromolar H_2_O_2_ in acidic media. Scientific Reports.

[8] RodríGuez Y, Ballester A, Blázquez ML, González F, Muñoz JA (2003). New information on the pyrite bioleaching mechanism at low and high temperature. Hydrometallurgy.

[9] Singer PC, Stumm W (1970). Acidic Mine Drainage: The rate-determining step. Science.

[10] Crundwell FK (2013). The dissolution and leaching of minerals. Hydrometallurgy.

[11] Fowler TA, Holmes PR, Crundwell FK (2001). On the kinetics and mechanism of the dissolution of pyrite in the presence of *Thiobacillus ferrooxidans*. Hydrometallurgy.

[12] Holmes PR, Fowler TA, Crundwell FK (1999). The mechanism of bacterial action in the leaching of pyrite by *Thiobacillus ferrooxidans*. Journal of the Electrochemical Society.

[13] Fowler TA, Holmes PR, Crundwell FK (1999). Mechanism of pyrite dissolution in the presence of *Thiobacillus ferrooxidans*. Applied & Environmental Microbiology.

[14] Luther GW (1988). Pyrite oxidation and reduction - molecular orbital theory considerations. Geochim. Cosmochim. Acta.

[15] Moses CO, Nordstrom DK, Herman JS, Mills AL (1987). Aqueous pyrite oxidation by dissolved oxygen and by ferric iron. Geochim. Cosmochim. Acta.

[16] Basson P, Gericke M, Grewar TL, Dew DW, Nicol MJ (2013). The effect of sulphate ions and temperature on the leaching of pyrite. III. Bioleaching. Hydrometallurgy.

[17] Chandra AP, Gerson AR (2010). The mechanisms of pyrite oxidation and leaching: A fundamental perspective. Surface Science Reports.

[18] Chandra AP, Gerson AR (2011). Redox potential (Eh) and anion effects of pyrite (FeS_2_) leaching at pH 1. Geochim. Cosmochim. Acta.

[19] May N, Ralph D, Hansford G (1997). Dynamic redox potential measurement for determining the ferric leach kinetics of pyrite. Minerals engineering.

[20] Sun H, Chen M, Zou L, Shu R, Ruan R (2015). Study of the kinetics of pyrite oxidation under controlled redox potential. Hydrometallurgy.

[21] Silverman MP, Ehrlich HL, Silverman MP (1964). Microbial formation and degradation of minerals. Advances in Applied Microbiology.

[22] Rohwerder T, Gehrke T, Kinzler K, Sand W (2003). Bioleaching review part A: Progress in bioleaching: fundamentals and mechanisms of bacterial metal sulfide oxidation. Applied Microbiology and Biotechnology.

[23] Sand W, Gehrke T, Jozsa PG, Schippers A (2001). (Bio)chemistry of bacterial leaching—direct vs. indirect bioleaching. Hydrometallurgy.

[24] Schippers A (2004). Biogeochemistry of metal sulfide oxidation in mining environments, sediments, and soils. Special Paper of the Geological Society of America.

[25] Schippers A, Jozsa P, Sand W (1996). Sulfur chemistry in bacterial leaching of pyrite. Applied & Environmental Microbiology.

[26] Schippers A, Rohwerder T, Sand W (1999). Intermediary sulfur compounds in pyrite oxidation: implications for bioleaching and biodepyritization of coal. Applied Microbiology & Biotechnology.

[27] Toniazzo V, Mustin C, Benoit R, Humbert B, Berthelin J (1999). Superficial compounds produced by Fe(III) mineral oxidation as essential reactants for bio-oxidation of pyrite by *Thiobacillus ferrooxidans*. Process Metallurgy.

[28] Tributsch H (2001). Direct versus indirect bioleaching. Hydrometallurgy.

[29] Sand W, Gerke T, Hallmann R, Schippers A (1995). Sulfur chemistry, biofilm, and the (in) direct attack mechanism—a critical evaluation of bacterial leaching. Applied Microbiology and Biotechnology.

[30] Vera M, Schippers A, Sand W (2013). Progress in bioleaching: fundamentals and mechanisms of bacterial metal sulfide oxidation—part A. Applied Microbiology & Biotechnology.

[31] Becker T, Gorham N, Shiers DW, Watling HR (2011). *In situ* imaging of *Sulfobacillus thermosulfidooxidans* on pyrite under conditions of variable pH using tapping mode atomic force microscopy. Process Biochemistry.

[32] Gehrke T, Telegdi J, Thierry D, Sand W (1998). Importance of extracellular polymeric substances from *Thiobacillus ferrooxidans* for bioleaching. Applied and Environmental Microbiology.

[33] Pisapia C, Humbert B, Chaussidon M, Mustin C (2008). Perforative corrosion of pyrite enhanced by direct attachment of *Acidithiobacillus ferrooxidans*. Geomicrobiology Journal.

[34] Crundwell FK (1998). The indirect mechanism of bacterial leaching. Mineral Processing & Extractive Metallurgy Review.

[35] Florian B, Noël N, Thyssen C, Felschau I, Sand W (2011). Some quantitative data on bacterial attachment to pyrite. Minerals Engineering.

[36] Liu H, Gu G, Xu Y (2011). Surface properties of pyrite in the course of bioleaching by pure culture of *Acidithiobacillus ferrooxidans* and a mixed culture of *Acidithiobacillus ferrooxidans* and *Acidithiobacillus thiooxidans*. Hydrometallurgy.

[37] Bellenberg S (2015). Manipulation of pyrite colonization and leaching by iron-oxidizing *Acidithiobacillus* species. Applied Microbiology and Biotechnology.

[38] Meyer H (1969). How oxidation affects selective flotation of complex sulphide ores. Canadian Metallurgical Quarterly.

[39] Ruan R (2013). Why Zijinshan copper bioheapleaching plant works efficiently at low microbial activity – Study on leaching kinetics of copper sulfides and its implications. Minerals Engineering.

[40] Ruan R (2011). Industrial practice of a distinct bioleaching system operated at low pH, high ferric concentration, elevated temperature and low redox potential for secondary copper sulfide. Hydrometallurgy.

[41] Yu JY, McGenity TJ, Coleman ML (2001). Solution chemistry during the lag phase and exponential phase of pyrite oxidation by Thiobacillus ferrooxidans. Chemical Geology.

[42] Hong P (2006). Structure analysis of 16S rDNA sequences from strains of Acidithiobacillus ferrooxidans. Journal of biochemistry and molecular biology.

[43] Bates ST (2011). Examining the global distribution of dominant archaeal populations in soil. International Society for Microbial Ecology Journal..

